# Temporal Processing in the Auditory System: Insights from Cochlear and Auditory Midbrain Implantees

**DOI:** 10.1007/s10162-012-0354-z

**Published:** 2012-10-17

**Authors:** Colette M. McKay, Hubert H. Lim, Thomas Lenarz

**Affiliations:** 1 Audiology & Deafness Research Group, School of Psychological Sciences, University of Manchester, Manchester, M13 9PL UK; 2 Departments of Biomedical Engineering and Otorhinolaryngology, University of Minnesota, Minneapolis, MN USA; 3 Department of Otorhinolaryngology, Hannover Medical University, Hannover, Germany

**Keywords:** cochlear implant, auditory midbrain implant, temporal resolution, loudness, temporal integration

## Abstract

Central auditory processing in humans was investigated by comparing the perceptual effects of temporal parameters of electrical stimulation in auditory midbrain implant (AMI) and cochlear implant (CI) users. Four experiments were conducted to measure the following: effect of interpulse intervals on detection thresholds and loudness; temporal modulation transfer functions (TMTFs); effect of duration on detection thresholds; and forward masking decay. The CI data were consistent with a phenomenological model that based detection or loudness decisions on the output of a sliding temporal integration window, the input to which was the hypothetical auditory nerve response to each stimulus pulse. To predict the AMI data, the model required changes to both the neural response input (i.e., midbrain activity to AMI stimuli, compared to auditory nerve activity to CI stimuli) and the shape of the integration window. AMI data were consistent with a neural response that decreased more steeply compared to CI stimulation as the pulse rate increased or interpulse interval decreased. For one AMI subject, the data were consistent with a significant adaptation of the neural response for rates above 200 Hz. The AMI model required an integration window that was significantly wider (i.e., decreased temporal resolution) than that for CI data, the latter being well fit using the same integration window shape as derived from normal-hearing data. These models provide a useful way to conceptualize how stimulation of central auditory structures differs from stimulation of the auditory nerve and to better understand why AMI users have difficulty processing temporal cues important for speech understanding.

## Introduction

Auditory midbrain implants (AMIs) are designed for electrically stimulating the neurons of the inferior colliculus (IC) to elicit sound sensations in patients who are profoundly deaf and are not suitable for cochlear implantation (CI) (e.g., those without a functional auditory nerve [AN] or implantable cochlea). The development of the AMI was based upon hypotheses that poor outcomes with the auditory brainstem implant (ABI) were related to tumor or surgical damage to important cells in the cochlear nucleus (CN) (Colletti 2006), and the theoretical ability to access frequency layers in the IC. Early studies with the first recipients of the AMI device (Lenarz et al. 2006a,b, 2007; Lim et al. 2007, 2008a,b, 2009) showed that patients gained benefits for speech understanding, when combined with lip-reading, that were similar to those generally observed for patients using ABIs for stimulation of the CN (McCreery 2008; Schwartz et al. 2008; Colletti et al. 2009). Although these outcomes are poorer than expected for CI users, it is possible that performance could be improved with development of more appropriate electrode array designs and processing strategies that are optimized for stimulation of the IC. The ability to detect envelope modulation in speech signals is important for speech understanding, and the ability to monitor intensity differences across frequency channels and over time is also crucial for dynamic spectral shape perception (Shannon et al. 1995). Thus, successful amplitude modulation and intensity coding in the electrical signal are important aims of signal processing for any auditory implant. The current study investigated the temporal processing abilities of the AMI recipients related to these auditory features, with the aim of gaining knowledge that would underpin future development of the device.

The perception of amplitude modulation and loudness has been studied extensively in CI users (Shannon 1992; Nelson et al. 1996; McKay and McDermott 1998; Zeng et al. 1998; Donaldson and Viemeister 2000; Galvin and Fu 2009; Chatterjee and Oberzut 2011; Fraser and McKay 2012). For electrical stimulation of the AN, central processing of intensity information over time can be usefully described using the same temporal integration (TI) model developed for normal acoustic hearing (McKay and McDermott 1998; McKay et al. 2003), assuming that the amount of neural activity in the peripheral nerve is the input information that the central auditory system relies upon in both cases.

The neural coding of stimulus intensity information in the CN and IC is more complex than in the AN. There are different types of neurons in these structures that play varying roles in the parallel processing of different stimulus features, and that have complex excitatory and inhibitory connections related to both afferent and efferent inputs (Cant and Benson 2003; Oliver 2005; McCreery 2008) Additionally, at the CN or IC level, the way intensity is neurally coded may diverge from a relatively simple ‘amount of activity’ code that can be applied as a first approximation in the AN (Ehret and Schreiner 2005; Young 2010). Therefore, when considering appropriate ways to electrically stimulate these structures to convey intensity information (including its temporal modulation), both the types of neurons activated and the appropriate temporal or spatial patterns of activation will be important. Although research in this area is important for both ABI and AMI users, the focus of this paper is on AMI users, to evaluate the need for alternative signal processing strategies in clinical use of the IC-based device.

In this study, a series of psychophysical experiments with three AMI users are described. The experiments explore the effects that different temporal parameters of electrical stimulation have on stimulus detection or on the perception of stimulus intensity and temporal modulation. The results are compared to those from similar previous experiments with CI users. The results from both types of implants are modeled using a phenomenological model of central auditory processing to gain insight into how central processing of intensity and temporal information differs between CI and AMI users.

## Subjects and equipment

Three AMI users participated in the experiments. All were adults who suffered from Neurofibromatosis type 2 (NF2) and had tumors removed prior to AMI device implantation at the Hannover Medical University (Germany). Table 1 provides information about each subject as well as their electrode array placement and clinical device settings. Note that, although the intended target for array placement was the central nucleus of the IC (ICC), this was not achieved in all cases. Electrodes for AMI-2 were near the ventral portion of the lateral lemniscus, those for AMI-3 were in the ICC, and those for AMI-5 were in the brachium of the IC. The midbrain location of the AMI array in each subject was determined from fused MRI and CT images that were superimposed on fixed human brain slices taken from the anatomical brain collection at the Department of Neuroanatomy in Hannover Medical University (Kretschmann and Weinrich 1992) and compared with detailed midbrain sections presented by Geniec and Morest (1971). Further details of this method have been previously published by Lim et al. (2007). For the purposes of this study, we will refer to the stimulated neural population as midbrain neurons. Subject identification codes for two subjects (AMI-2 and AMI-3) are identical to those in previous reports (see Lim et al. 2009 for a review), where more details are presented for the surgical approach and array placement. AMI-2 could only participate in experiment 1 for health reasons related to the NF2 condition. This study was conducted in accordance with ISO 14155 (International Standard for Clinical Investigation of Medical Devices) and follows the Good Clinical Practice guidelines. Medical Ethics Committee and Competent Authority written approvals according to national laws were obtained and the patients signed informed consent forms prior to AMI implantation and testing.

**TABLE 1:** Details of the subjects

Subject	Electrode insertion site	Clinical strategy	Electrode used in experiments	Clinical T [and C] (CL)
AMI-2	Lateral lemniscus	SPEAK 250 Hz	18	147 [164]
AMI-3	Central nucleus of IC	SPEAK 250 Hz	6	126 [157]
AMI-5	Brachium of IC	ACE 250 Hz	7	134 [196]

Psychophysical experiments were carried out using ImpResS software, interfaced with the implant via a SPEAR processor (see Acknowledgements). The software defined the stimulus parameters, ran the experimental procedure, and collected subject responses via a response box. The processor sent the coded instructions for each stimulus directly to the implanted electronics via a radio frequency link.

## Temporal integration model

Many temporal effects in normal hearing (loudness variation, masking, temporal resolution) can be well described using a phenomenological TI model, whereby the peripheral nerve activity in the auditory system is summed in a sliding TI window that weights the activity occurring at different times, and a decision device acts upon the output of the integration window. Early versions of the acoustic model (e.g., Moore et al. 1988) acted upon the stimulus intensity itself, leading to the integration window being frequency and intensity dependent. However, later work (Oxenham and Moore 1994; Moore et al. 1996; Plack and Oxenham 1998; Oxenham 2001; Plack et al. 2002) has shown that, if the nonlinear processes within the cochlea are first taken into account, the integration window can be considered a linear smoothing process following cochlear activation. Plack et al. (2002) argued that the linear TI window should act upon the *intensity* of basilar membrane vibration, which in turn may be linearly related to AN firing rate (Muller et al. 1991). Thus, it is plausible that the TI window determined by these later studies could be applied directly to AN activity evoked by CI stimulation. Indeed, a version of the TI model has been used to predict the effects of interpulse intervals and amplitude modulation on loudness in CI users (McKay and McDermott 1998; McKay and Henshall 2010). Two earlier models acted upon the stimulus waveform. Shannon (1989) accounted for the effect of pulse rate and pulse width on thresholds in CI users using a model that incorporated a TI window that acted upon a power function of the stimulus waveform. A model by Carlyon et al. (2005) accounted for the effect of frequency of sinusoidal stimuli and interphase gaps in low-rate pulsatile stimuli using a model that incorporated a TI window that acted upon a low-pass filtered stimulus waveform.

The TI window used in this paper follows the form used by recent papers in the acoustic hearing field (Oxenham and Moore 1994; Oxenham 2001; Plack et al. 2002):

1 $$ \matrix{ {W(t)=\left( {1-w} \right)\exp \left( {{t \left/ {{{T_{\mathrm{b}1}}}} \right.}} \right)+w\exp \left( {{t \left/ {{{T_{\mathrm{b}2}}}} \right.}} \right),\,t < 0} \\ {W(t)=\exp \left( {{-t \left/ {{{T_{\mathrm{a}}}}} \right.}} \right),\,t\geqslant 0,} \\ }<!end array> $$

where *W*(*t*) is the weight applied at time *t* relative to the peak of the function, *T*_a_ and *T*_b1_ together define the short time constant associated with temporal resolution, *T*_b2_ defines a longer tail of the window associated with forward masking and the effect of stimulus duration, and *w* is the weighting of the long versus short time constants. Oxenham (2001) derived the integration window shape to best fit forward masking data in normally hearing listeners: the best-fitting values of the parameters were *T*_a_ = 3.5 ms, *T*_b1_ = 4.6 ms, *T*_b2_ = 16.6 ms, and *w* = 0.17. These parameter values are used here to model the previously published CI data.

**FIG. 1:**
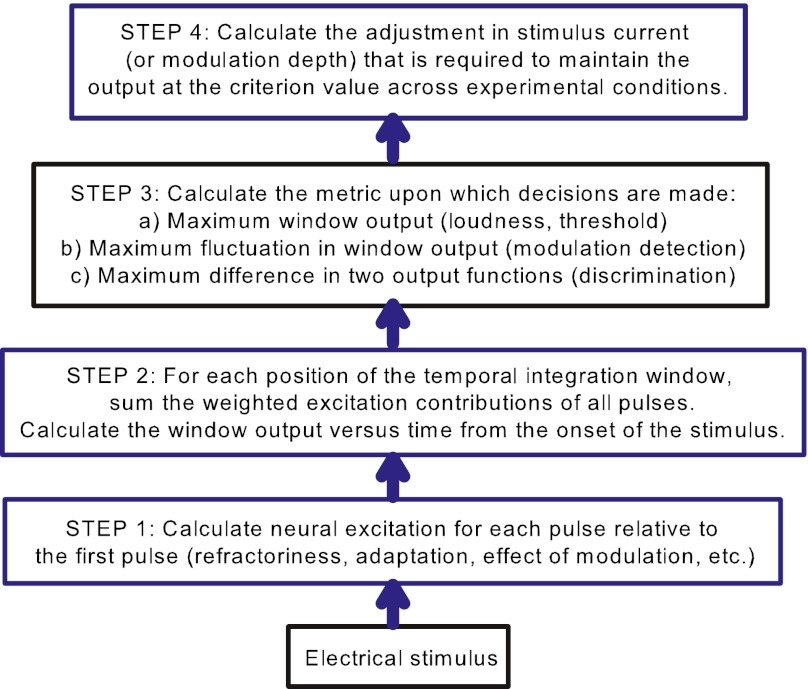
A schematic of the TI model, showing the steps used to predict the data in the four experiments.

Figure 1 illustrates the steps used to model the outcome of the experiments in this paper. First, a transformation was applied to derive the neural excitation evoked by each pulse relative to a fixed reference (usually the excitation to the first pulse). The parameters of this transformation, which takes into account effects such as refractoriness and adaptation, were adjusted to be consistent with known neural behavior and could be fitted in the modeling process. This part of the model was different for each experiment, and is described in more detail within the “TI model” section at the end of each experimental section below. In the second step, the neural excitation for each pulse in the stimulus became the input to the TI window. The output of the window was the weighted sum of all stimulus pulses as per Eq. 1. A function of window output versus time was calculated by moving the peak of the window (*t* = 0) along the length of the stimulus in increments corresponding to the stimulus pulse intervals. The third step involved extracting the relevant criterion of window output specific to each experiment. Stimuli of equal loudness were assumed to have an equal *maximum integrator output*. Detection threshold was assumed to be the stimulus level that evoked a fixed criterion maximum window output (assuming that internal noise was constant for the experiment). In masking experiments, it was assumed that a signal was detected when the signal-to-noise (or signal-to-masker) ratio at the output of the integrator was a fixed criterion ratio (*k* dB). Similarly, detection of modulation was assumed to occur when the integrator output fluctuated by a criterion ratio. Finally, to predict the way that the stimulus level (or modulation depth) must be changed across experimental conditions to maintain a fixed criterion value (fixed maximum window output, fixed difference in window output, or fixed fluctuation of window output), a model of how neural excitation changes with current level was applied. This is an unknown relationship that will vary across subjects and electrodes. Here, the slope of this function in log/log units was denoted as *S*. That is, it was assumed that, over small ranges of current, the current versus neural excitation function could be described as a power function with exponent *S*. For experiments in which the current varied over only a small range for different conditions, *S* was assumed to be constant across the conditions for a particular subject and overall level. *S* was allowed to differ between subjects and between absolute reference levels in the same subject. McKay et al. (2003) showed that the current-to-loudness function in CI users (again on log/log scales) had a constant slope for low current levels, but an increasing slope with level for currents above a subject-specific kneepoint. If loudness can be viewed as a power function of neural activity, this suggests that *S* would also be relatively constant at low current levels and increase with level at higher current levels.

In the following experiments, the effects of temporal stimulus parameters on loudness, detection and discrimination thresholds, and forward masked thresholds were investigated. The ability of the TI window model to explain analogous, previously published, CI data, and the way that the model would need to be amended to account for the AMI data, were investigated. The three blue boxes in Figure 1 represent steps where fitting parameters were used. For CI users, the TI window parameters in step 2 were fixed at the values detailed above that were derived from normal-hearing data.

For AMI subjects, the effects of changing the TI window parameters in step 2 were also investigated, as it would not be expected that activity of midbrain neurons would be centrally processed in the same way as activity of the AN. In the AMI case, there were generally too many parameters to independently fit them all to the data. Instead, a more general investigative approach was used, to evaluate how varying the different parameters changed the pattern of prediction. Since the TI model is phenomenological, it can make no claim to illuminate neural mechanisms. However, since the AMI sound processors (and hence, stimulus patterns) used clinically are identical to those used for CIs, the results of these experiments provide insights into the perceptual difficulties encountered by current AMI users.

## Experiment 1: interpulse intervals

### Rationale

McKay and McDermott (1998) showed that, for CI patients, the effect of interpulse interval (IPI) between a pair of pulses on loudness and detection threshold was consistent with the combined effects of two factors: reduced neural response to the second pulse due to refractory effects, and TI of neural activity from the two pulses within an exponential two-sided window of equivalent rectangular duration (ERD) of 7 ms. The differences between subjects and stimulus levels in the shape of the current-for-equal-loudness versus IPI functions could be modeled by variation in the proportion of ‘available’ neurons that fired on the first of each pulse pair, and their refractory recovery time (see “TI model” section). Experiment 1, therefore, repeated the experiment performed by McKay and McDermott (1998) with AMI users to determine how the effect of IPI in pulse pairs on loudness and threshold judgments differed from that of CI users.

### Stimuli and procedures

Pairs of cathodic-leading biphasic pulses were delivered to a single midbrain electrode at a repetition rate of 100 Hz. Note that McKay and McDermott (1998) used a 50-Hz repetition rate, but AMI-2 could not hear stimuli with 50 Hz rate because of a steep increase in threshold at low rates. All subjects used monopolar mode (MP1 + 2) and phase durations of 100 μs. Interphase gaps of 45 μs were used for AMI-2 and AMI-3 and 8 μs for AMI-5. The IPI between the onsets of the two pulses in each pair was varied between the smallest possible (262.5 μs for AMI-2 and AMI-3, and 225.5 μs for AMI-5) and 5 ms (at which point the stimulus became a steady 200-Hz pulse train). Electrodes 18, 6, and 7 were used for AMI-2, AMI-3, and AMI-5, respectively. The choices of electrode and pulse timing parameters were determined by selecting clinically used electrodes with unnoticeable side effects and the pulse parameters used in the subjects’ clinical processor. In addition to the pulse-pair stimulus, a single-pulse-per-period pulse train with a rate of 100 Hz was used as a reference stimulus for loudness comparisons. Current was controlled in current levels (CLs), which correspond to steps of 0.1569 dB.

Detection thresholds for all stimuli (reference and pulse-pair) were determined using an adaptive three-interval forced-choice (3IFC) procedure, in which one randomly chosen interval out of three contained the stimulus. Level was reduced after two correct responses and increased after every incorrect response (converging on 71 % correct). The step size for AMI-2 and AMI-3 was 4 CL for two reversals and 2 CL for the six remaining reversals. Step size for AMI-5 was 4 CL for all reversals as she had a very shallow loudness growth with level. Thresholds were calculated as the average CL at the final six reversals. Each threshold was measured at least twice and averaged. To monitor any changes in threshold throughout the sessions, the reference stimulus (100-Hz pulse train) threshold was re-determined throughout the sessions. In all cases presented here, this threshold was stable across measurements. All instances of this threshold were averaged for the subsequent data analysis.

The pulse-pair stimuli were loudness balanced with the reference stimulus (100-Hz pulse train) at a comfortable loudness. First, the reference stimulus was set at a comfortable loudness by the subject. Then an adaptive two-interval forced-choice (2IFC) task was conducted in which the reference and test stimuli were presented in random order and the subject responded with which interval contained the louder stimulus. The CL of the test stimulus was incremented or decremented based on the answer to each trial (asymptoting to the CL for 50 % louder response). The step size, number of turns and number averaged were the same as in the detection threshold task for each subject. Four balances were achieved for each stimulus, two with the test stimulus being adjusted and two with the reference stimulus being adjusted. The four balances were averaged to find the current reduction (compared to the current in the reference stimulus) required for the pulse-pair stimulus to be the same loudness as the reference stimulus.

## Results and discussion

**FIG. 2:**
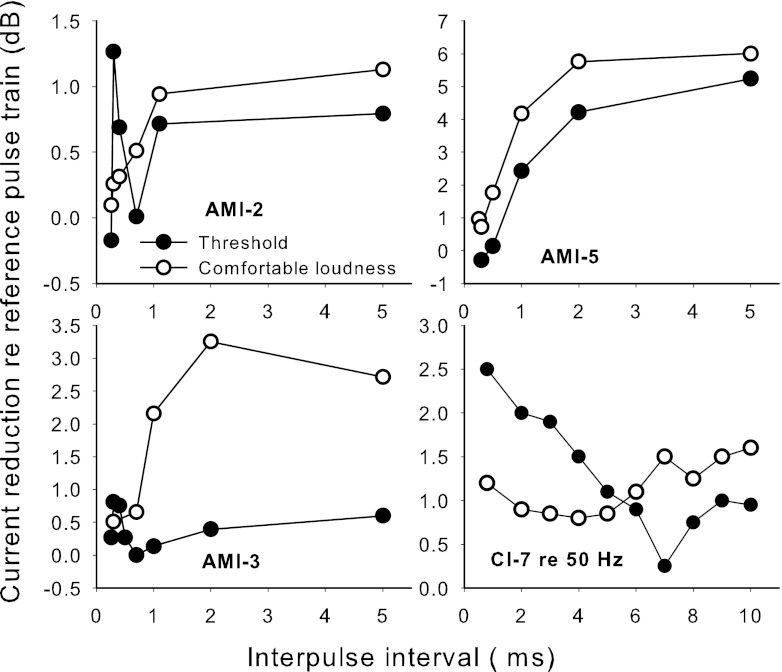
Results of experiment 1. For each AMI subject the current reduction of the pulse-pair stimulus relative to the 100-Hz reference pulse train to make it the same loudness at comfortably loud level or at threshold is shown. In the lower right panel are some example CI data (subject 7) from McKay and McDermott (1998). The reference pulse train had a 50-Hz pulse rate for the CI data.

The results of experiment 1 are shown in Figure 2. At the comfortably loud level, the results of all three AMI subjects showed a similar pattern in which the current adjustments required to make the pulse-pair stimuli the same loudness as the reference were close to zero for the smallest IPIs and increased with increasing IPI up to around 2 ms. The shapes of the functions differed significantly from those of CI subjects in the study of McKay and McDermott (1998), which varied with subject and level and included exponentially falling, non-monotonic, or flat current adjustments with increasing IPI, but were never substantially increasing with IPI like the AMI data. The lower-right panel in Figure 2 shows example data for one of the CI subjects (subject 7) from that paper. The AMI data indicate that: (a) integration of neural activity from the second pulse with that from the first pulse is not occurring for small IPIs and/or the second pulse is producing negligible neural response for small IPIs; and (b) the contribution from the second pulse shows maximum recovery after 2 ms, in contrast to CI subjects for whom the average 50 % recovery time was modeled to be around 7 ms.

The AMI detection threshold functions in Figure 2 showed a pattern similar to those for comfortable loudness, except that there was an unexpected transient peak for AMI-2 and AMI-3 at a very small IPI (300 μs). Efforts were made to rule out artifact as an explanation for these peaks. More data were collected for nearby IPIs (more IPIs and repeat measurements) to rule out variance in the measurements as an explanation. The output of a test implant was examined on an oscilloscope to ensure that the second pulse was actually present when the IPI was the smallest (262.5 or 265.5 μs). The negligible effect at the smallest IPI ruled out charge summation as an explanation for the peak, as charge summation would lead to the smallest IPI producing a lower threshold than an IPI of 300 μs. It seems likely, then, that the peak reflects a property of the neural response specific to midbrain neurons. It is interesting to note that the peak occurred in the two subjects who used a longer interphase gap of 45 μs. If the gap between the offset of the second phase of the first pulse and the onset of the first phase of the second pulse were also 45 μs, the IPI would be 290 μs, close to where the peak occurred. Thus, if both phases were excitatory, this condition would evoke neural responses that contained sequences of three equal intervals. The peak introduces doubt that the recovery-like functions seen in Figure 2 are due solely to refractory behavior of the stimulated midbrain neurons. Instead, both the recovery shape and the peak could reflect the response pattern of neurons higher in the auditory system, which may modulate their behavior based on input spike intervals or patterns from midbrain neurons.

### TI model

The effect of IPI was modeled similarly to McKay and McDermott (1998), except that slightly updated TI parameters were used for a hypothetical CI user, as explained in the description of Eq. 1. It was assumed that stimuli that were equally loud (or at detection threshold) led to equal *maximum* output of the TI window as it moved across the stimulus duration. Following the steps in Figure 1, first, the magnitude of the neural response to the second of each pair of pulses (*E*_2_) relative to the first (*E*_1_^pp^) as a function of *IPI* was calculated from the following equation:

2 $$ {E_2}=E_1^{\mathrm{pp}}\left( {1-\frac{R}{{\left( {1+{{\mathrm{e}}^{{{{{\left( {\mathrm{IPI}-{t_0}} \right)}} \left/ {D} \right.}}}}} \right)}}} \right), $$

where *R* is the proportion of available neurons (i.e., the proportion of those that were above their absolute threshold for the particular stimulus current) that fired to the first pulse, and hence were affected by refractory recovery for the second pulse, and *t*_0_ is the recovery time constant. The divisor *D* governed the slope of the recovery function, and is related to the standard deviation of the individual neural recovery times: if all neurons recovered at a very similar time the function would be very steep, and, conversely if the recovery times were relatively spread, the function would have a more gradual slope. It was assumed that the repetition period was sufficiently long for complete refractory recovery after each pulse pair, so that *E*_1_^pp^ and *E*_2_ were the same in each period along the stimulus. For the AMI users, *R*, *t*_0_, and *D* were fitting parameters, and for the hypothetical CI user, *t*_0_ and *D* were fixed at values representing typical values (7.3 and 0.8, respectively) found for CI users by McKay and McDermott (1998).

**FIG. 3:**
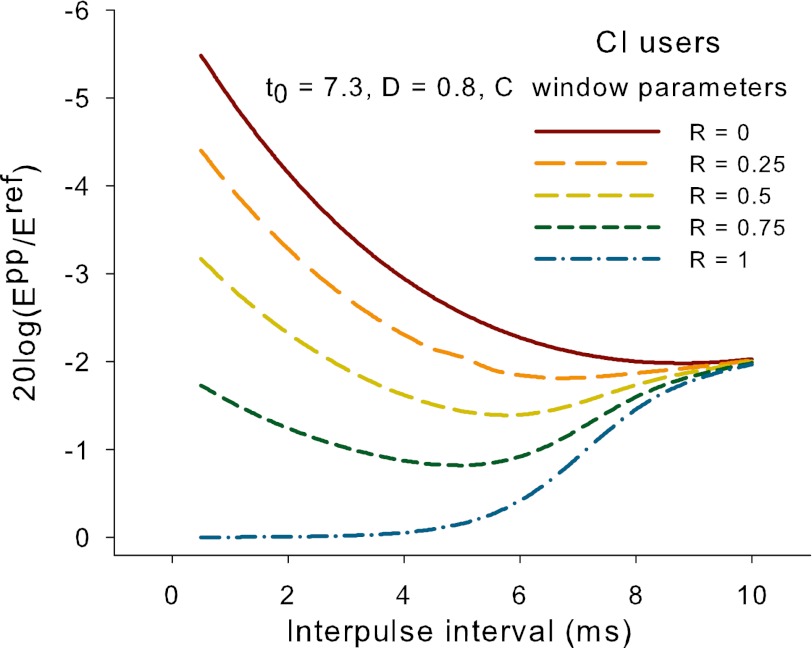
The effects of different *R* values on the model output are shown, using the CI window parameters and average *t*_0_ and *D* values from the McKay and McDermott (1998) paper. The CI data from that paper showed variable function shapes that were consistent with *R* values between 0.25 and 0.9.

In the second step, the maximum TI window output was calculated for the reference pulse train (as a function of *E*_1_^ref^, the excitation for each pulse in that stimulus — again assuming no refractory effects between the periods), and this became the criterion window output for threshold or comfortable loudness that was applied to the pulse-pair stimuli. Similarly, the maximum TI window outputs for the pulse-pair stimuli were calculated as a function of *E*_1_^pp^, using Eq. 2 to derive the excitation to the second pulse relative to the first. Equating the maximum window output for the reference and pulse-pair stimuli then allowed the ratio *E*_1_^pp^/*E*_1_^ref^ to be calculated in dB. Figure 3 shows this ratio versus IPI for our hypothetical CI user for values of *R* ranging from zero to one. It can be seen that *R* influences the shape of the function, which varies from exponentially falling curve for *R* close to 0, to a rising curve for *R* equal to 1, and a non-monotonic curve for in-between values of *R*. The different shapes are a result of the trade-off between refractory recovery and TI. Finally, the last step converted the *E*_1_^pp^/*E*_1_^ref^ ratio (in dB) to the current change (in dB) applied to the pulse-pair stimulus to equate its loudness to the reference stimulus by dividing by the exponent *S*. The functions of current change versus IPI were thus scaled versions of the functions of 20 log(*E*_1_^pp^/*E*_1_^ref^) versus IPI. Note that vertical axis in Figure 2 is defined as ‘current reduction’ and hence has an opposite sign to the graph shown in Figure 3.

The CI data presented in McKay and McDermott (1998) varied considerably between subjects and levels, and showed all the function shapes shown in Figure 3 that were consistent with values of *R* ranging from 0.3 to 0.9. However, none of the functions showed a notable increase of current adjustment with increasing IPI at short IPIs. For the CI subject shown in Figure 2, the fitted parameters of [*R*, *t*_0_, S] were [0.88, 6.9, 0.73] at comfortably loud level and [0.4, 7.8, 1.4] at threshold. Although the mean *R* and *t*_0_ across the CI group did not vary significantly with level, the mean value of *S* increased from 1.4 at threshold (range 1.1–1.9) to 3.3 at comfortably loud level (range 0.7–6.1).

**FIG. 4:**
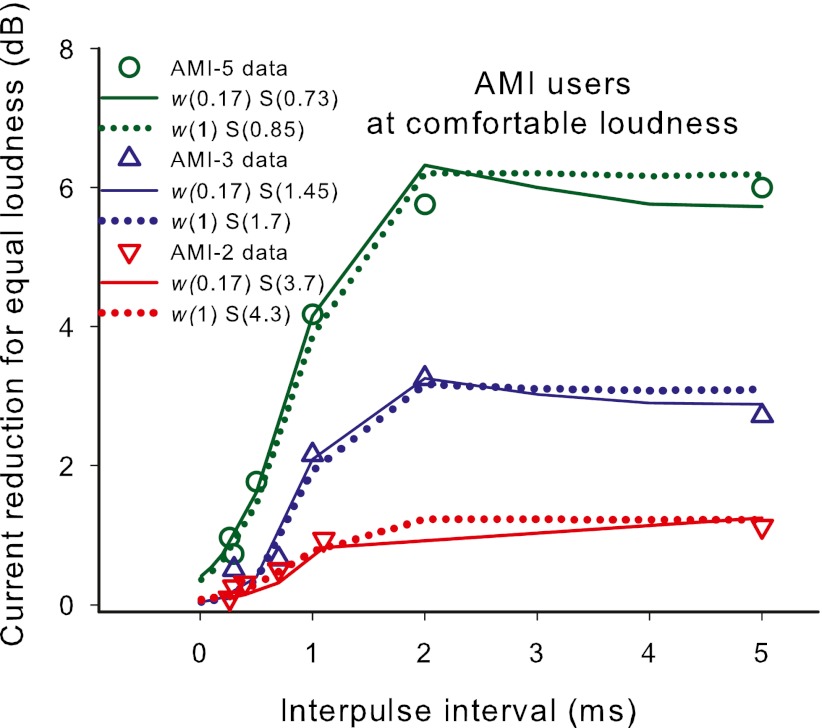
Model outputs fit to the data of the three AMI users at comfortable level only. The *solid lines* show model outputs using the TI window shape as defined by recent acoustic data from normally-hearing subjects, and the *dotted lines* show model outputs when the weighting (*w*) of long to short time constants was increased to 1 (i.e., no short time constant). The fitted values of S shown in the panels are the slopes (on log/log scales) of the current to neural excitation function. Note that the data in Figure 2 and in this figure are defined as ‘current reduction’ and hence the vertical axis has an opposite sign to the graph shown in Figure 3.

Only the AMI data at comfortable level were modeled, since this simple model could not predict the transient peak in the threshold data observed for two of the subjects. However, the threshold data could be similarly modeled if the peaks were ignored, as was done later for experiment 3. The comfortable level data were first modeled by assuming that the TI window was the same as for CI subjects but the effect of IPI on the neural activity due to the second pulse of each pulse pair (parameters of Eq. 2) differed from that in CIs. Since the data showed little influence of the second pulse on loudness for very small IPIs (where TI would have the greatest influence) the *R* value was assumed to be 1 (i.e., all midbrain neurons that were above their absolute threshold for that current level fired on the first pulse, and hence were not available until they recovered from their refractory state — see Figure 3 to see effect of *R* = 1 in the CI model). To fit the data, the recovery time constant (*t*_0_) had to be reduced to 1 ms, and the divisor D was reduced to 0.3 (for AMI-2 and AMI-5) or 0.2 (for AMI-3). These changes (increased *R* with decreased *t*_0_ and D) are consistent with each other, and with the proposition that most activated midbrain neurons were activated well above their thresholds. If this were the case, there would be few neurons available to respond to the second pulse within the refractory period (large *R*), and the refractory period would be short (small *t*_0_) and would vary less among the activated neurons (small *D*). The solid lines in Figure 4 show the predicted effect of IPI for each AMI subject at comfortable level, using the final fitting parameter *S* to scale the data.

It can be seen from Figure 4 that the AMI data can be fit reasonably well by assuming the same TI window as CI subjects, but using an altered neural recovery function to calculate the input to the window. However, since the TI window has its maximum influence at short IPIs, for which AMI users seemingly show very little neural activity to the second pulse, this experiment was rather insensitive to TI window parameters. Thus, given the input recovery function as modeled above, changes to the TI window parameters did not appreciably change the goodness of fit of the model to the data. To illustrate this point, the dotted lines in Figure 4 are fitted using the same neural excitation input function as the solid lines but with the weighting factor in the TI window, *w*, set to 1 instead of 0.17, leaving only the longer time constant.

In summary, the results of experiment 1 are consistent with the midbrain neurons having a different refractory behavior from that of the AN neurons, with very little or no activity (or contribution) evoked by a pulse that follows another within 2 ms. In contrast, the CI data showed a variable but always significant influence of the second pulse for small IPIs. Thus, the AMI data suggest a lack of short-term TI that could be solely the result of refractory behavior in the midbrain, but could also be consistent with changes to the TI window parameters combined with the strong refractory effects.

## Experiment 2: amplitude modulation detection

### Rationale

The ability to hear the amplitude modulations in a speech signal is crucial to speech understanding. There are two factors in modulation detection to consider: the sensitivity to modulation (related to the modulation detection threshold [MDT] at low modulation frequencies) and the temporal resolution (related to the cut-off modulation frequency of the low-pass-shaped TMTF). Temporal modulation sensitivity is highly correlated with intensity discrimination in CI users (Donaldson and Viemeister 2000; Galvin and Fu 2009) and in some studies has been shown to correlate with speech understanding in CI (Fu 2002) or ABI users (Colletti and Shannon 2005). Temporal resolution (cut-off modulation frequency) has been shown to be largely similar in CI users and normally hearing listeners (Fraser and McKay 2012), which would be expected if the processing of modulation frequency occurs in the central auditory system. Temporal resolution is directly limited by the shape and width of the central TI window, which has the effect of smoothing the faster modulations in the peripheral neural response. Fraser and McKay (2012) showed that limiting overall loudness cues in the modulation detection task led to steeper TMTFs in CI users at higher modulation frequencies than those measured without limiting loudness cues (the latter applying in most previous studies). Thus, appropriate loudness balancing and the use of stimulus current jitter are necessary to limit the confounding effects of modulation on overall loudness for each individual (McKay and Henshall 2010). Several studies have shown that higher carrier rates in CI stimulation can lead to poorer modulation detection (Galvin and Fu 2005; Pfingst et al. 2007; Fraser and McKay 2012; Green et al. 2012). Therefore, in this experiment, TMTFs for AMI-3 and AMI-5 were measured using different carrier rates while using a method that limited the use of loudness cues in the task.

### Stimuli and procedures

Reference unmodulated stimuli were pulse trains with rates of 300, 600, and 1,200 Hz, and duration of 500 ms. The same electrodes and pulse parameters (mode, phase duration, interphase gap) were used as in experiment 1. The modulated stimuli had the same carrier rate and other parameters as the reference stimulus. Sinusoidal modulation of the current parameter was used. The modulation depth was defined as the peak-to-valley current level difference (in CL) and later converted to the 20 log[*m*] notation for analysis, where m is the modulation index. A range of modulation depths was used (up to a maximum of 50 CL) and a range of modulation frequencies from 5 to 100 Hz. When adjusting the level of the modulated stimulus, all pulses in the pulse train were incremented by equal numbers of CLs. To avoid unwanted modulations due to aliasing and under-sampling of the modulation depth, all modulation frequencies were submultiples of the carrier rate and all modulated stimuli started with the maximum (peak) current.

First, the reference stimulus with carrier rate 600 Hz was set to a level deemed comfortable by the subject. Then a modulation frequency was selected and for each modulation depth to be tested, the corresponding modulated stimulus was loudness balanced with the reference stimulus using the balancing procedure described in Experiment 1. Following loudness balancing, the ability of the subject to discriminate each modulated stimulus (of different modulation depths) from the reference stimulus was measured using a 3IFC discrimination task, in which one interval, randomly selected, contained the modulated stimulus and the remaining two intervals contained the reference stimulus. To limit the use of any residual loudness cues after loudness balancing, a random level jitter of ±2 current steps (AMI-3) or ±4 current steps (AMI-5) was applied in each interval. Finally, a psychometric function was constructed of percent-correct identification (averaged over 25 trials) versus modulation depth, from which the MDT was calculated as the interpolated modulation depth for 70 % correct. The whole procedure (loudness balances and 3IFC tasks) was then repeated for other modulation frequencies to complete the TMTF for 600 Hz carrier rate.

The reference unmodulated stimuli for 300 and 1,200 Hz carrier rates were then set to the same loudness as the 600-Hz reference stimulus using the same loudness balancing task as described above. TMTFs were obtained for these carrier rates using the same procedure as for the 600-Hz carrier rate.

## Results and discussion

**FIG. 5:**
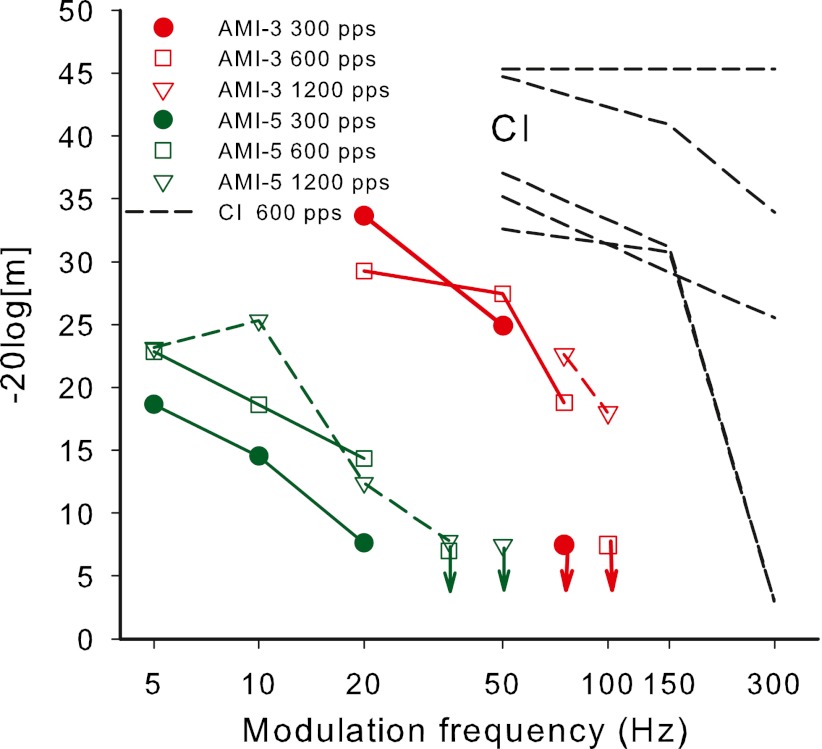
Results of experiment 2, showing TMTFs of AMI-3 (*red*) and AMI-5 (*green*) along with the TMTFs of five CI users re-plotted from Fraser and McKay (2012). The measurement techniques were identical in the two studies. *m* is the modulation index, where *m* = 1 corresponds to 100 % modulation (20 log[*m*] = 0). The symbols with *downward arrows* indicate conditions in which the subject could not detect the largest modulation depth used of 50 CL.

The TMTFs of AMI-3 and AMI-5 are shown in Figure 5, along with TMTFs from five CI subjects using a carrier rate of 600 Hz at a comfortably loud level that are re-plotted from Fraser and McKay (2012). The CI data were measured using the same techniques, including those to limit overall loudness cues, as the AMI data and are therefore directly comparable. Very poor temporal resolution was shown by both AMI subjects, with AMI-3 not being able to detect modulations above 75–100 Hz and AMI-5 not above approximately 20 Hz. In contrast all the CI subjects showed MDTs that did not become appreciably poorer until at least 150 Hz. It can be seen that AMI-3 had a similar maximum sensitivity (30–35 dB at 20 Hz) to that of the three CI users with poorer sensitivity (~30 dB at 50 Hz) but AMI-5 had very poor maximum sensitivity (<25 dB at 5 Hz) compared with both AMI-3 and the CI subjects.

If modulation detection is determined by modulations in the TI window output, the poor temporal resolution shown by AMI data would suggest that the TI window is much wider than in CI subjects or normally hearing subjects, leading to a smoothing of fluctuations at the output of the window. Alternatively, noisiness in the neural response could lead to very poor modulation detection, by inducing random fluctuations in the TI window output.

It is interesting to note that MDTs improved for higher carrier rates, especially at the higher modulation frequencies, for both AMI subjects. In contrast, higher carrier rates in CI subjects caused a small but significant reduction in modulation detection efficiency (Galvin and Fu 2005; Pfingst et al. 2007; Fraser and McKay 2012). The mechanism for the effect of carrier rate is unclear for either device, provided that the carrier rate is high enough to adequately sample the modulation. Fraser and McKay (2012) postulated that higher carrier rates in CI subjects may lead to increased noisiness in the AN response, which could mask the stimulus modulations. Additionally, interactions between the slope of the current to neural excitation function and carrier rate may influence the effect of carrier rate on modulation detection, especially in CI users, where the higher carrier rates would be at lower current levels. This is supported by the results of Fraser and McKay (2012) and Green et al. (2012) who showed that the effect of carrier rate is greatly reduced when the modulation depth is expressed as a proportion of the dynamic range. Regardless of the reason for the effect of carrier rate in CI users, it is apparent that AMI and CI users show opposite effects of carrier rate.

### TI model

**FIG. 6:**
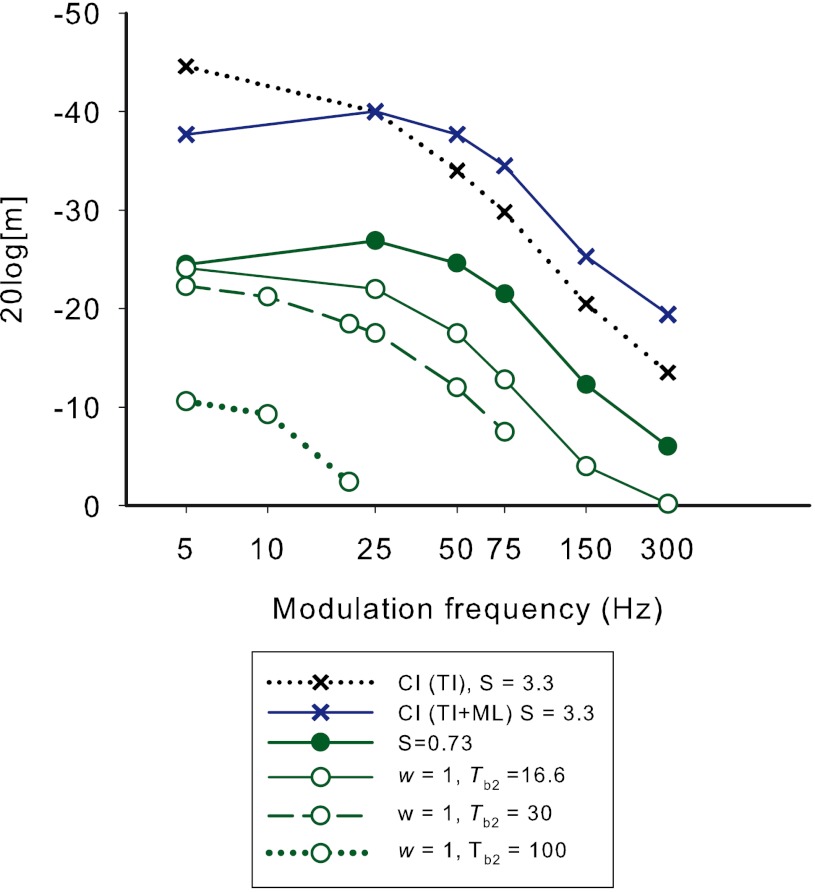
Model predictions of the effect of successive changes to the TI window shape on TMTFs. The *black crosses* represent the stimulus modulation depths that produce a fixed TI window output modulation depth of 0.33 dB using the parameters in Eq. 1. The *blue crosses* show the predicted effect of adding a multiple looks model to the prediction. The *solid circles* show the effect of decreasing the *S* value from 3.3 to 0.73, representing a change in S from the average CI value to that fitted for AMI-5 at comfortable level in experiment 1. The *open circles* show the successive effects of changing the weighting parameter (*w*) in the TI window to 1 (i.e., removing the short time constant) and setting the longer time constant (*T*_b2_) to 16.6, 30 or 100 ms.

To model modulation detection by the hypothetical CI user, it was first assumed that detection would occur when the TI window output modulation depth (OMD — ratio of peak to valley outputs in dB) was a fixed criterion value. For a carrier rate of 600 Hz, the TMTF was then modeled by finding the stimulus modulation depth for each modulation frequency that evoked this criterion OMD. First, the excitation evoked by each pulse relative to that evoked by the pulse with maximum current was calculated for different stimulus modulation depths using the relationship between current and excitation (i.e., using the exponent *S*). It was assumed that the modulation depth of the neural response (in dB) was not influenced by refractory effects (i.e., the exponent *S* governed the modulation depth in dB regardless of the average neural response). For the hypothetical CI user the average *S* value at comfortable level (3.3) from McKay and McDermott (1998) was used. The OMD, which was relatively constant across the stimulus, was then calculated from the function of TI window output versus time. From the graph of stimulus modulation depth versus OMD for the lowest modulation frequency (5 Hz), a criterion OMD (0.33 dB) was selected that corresponded to the typical stimulus modulation depth (−45 dB in 20 log[*m*] units) at MDT in CI users at low modulation frequencies. Next the stimulus modulation depths that resulted in the same criterion OMD for the higher modulation frequencies were calculated to derive the TMTF. The black crosses (dotted line) in Figure 6 show the resultant TMTF prediction for CI users. It can be seen that, although the prediction has a low-pass characteristic like the CI data in Figure 5, the slope for low modulation frequencies (<150 Hz) is generally steeper than the data.

The predicted TMTF can be improved by including the effects of multiple looks (Fig. 6, blue crosses, solid lines). Donaldson et al. (1997) showed that the effects of duration on detection threshold for 100-Hz pulse trains could be modeled by assuming that the ideal observer is able to store and use multiple ‘looks’ of the stimulus (Viemeister and Wakefield 1991) to improve detection: the performance (*d*′_*n*_) for *n* ‘looks’ compared to that for one look (*d*′_1_) is given by:

3 $$ d_n^{\prime }=\sqrt{{nd_n^{\prime }}} $$

In the case of modulation detection, each ‘look’ can be seen as a period of the modulation (in each of which the TI window OMD can be estimated). Therefore the number of looks available to the subject is the number of modulation periods contained in the stimulus duration. Each doubling of modulation frequency increases the number of looks available by a factor of 2, leading to an increase in performance of a factor of $\sqrt{2}$. If an assumption is then made that the performance (*d*′) is proportional to OMD, then it can be predicted that, to maintain a constant performance (i.e., threshold detection) as modulation frequency increases, the criterion OMD for threshold would decrease by a factor of ${c \left/ {{\sqrt{n}}} \right.}$, where *c* is a patient-specific constant. The blue crosses in Figure 6 show the predicted TMTF amended for multiple looks, while keeping the threshold at 25 Hz the same as before and using a constant of *c* = 1 and a maximum number of looks that could be stored limited to 40.

The poorer modulation detection of AMI subjects compared to CI subjects could be potentially due to several factors. The exponent, *S*, directly influences the MDTs: for a smaller *S*, a larger current modulation would be needed to achieve the same OMD. The filled green circles in Figure 6 show how the predicted TMTF would change when reducing *S* from 3.3 (blue crosses) to 0.73, a value that was fitted to the data for AMI-5 at comfortable level in experiment 1. The fact that *S* increases with absolute current level in CI users (McKay and McDermott 1998) may at least partially explain why MDTs become better as level is increased in those subjects. Similarly different values of *S* among subjects may contribute to differences in MDT, and may partially explain the difference in MDTs between AMI-3 and AMI-5 (see *S* values in experiment 1). However changes in *S* produce only a change in sensitivity, shifting the TMTF vertically and having little effect on the cut-off frequency. Therefore, to predict the poor temporal resolution (low cut-off frequency) of AMI users, it was necessary to increase the TI window duration.

Both the TI window weighting factor, *w*, and the time constants, affect the predicted temporal resolution. If the longer time constant is more dominant (larger *w*), then the OMD will become smaller for the higher modulation frequencies, leading to lower cut-off frequencies. The open circles in Figure 6 show the predicted change in TMTF from the closed circles when w is changed to 1 instead of 0.17 (i.e., no short time constant). Three predictions for *w* = 1 are shown, with increasing *T*_b2_. The first (solid line) has *T*_b2_ = 16.6, the value used for CI users, the second (dashed line) has *T*_b2_ = 30 ms, and the third (dotted line) has *T*_b2_ = 100 ms. It is seen that as *T*_b2_ increases, the cut-off frequency decreases with even 5-Hz modulations being difficult to hear for *T*_b2_ = 100 ms.

It was not possible to uniquely determine parameters in the TI model to fit the AMI data since many of the parameter changes (*S*, *w*, *T*_b2_) have dependent effects on the TMTF. Also, factors other than TI window shape may be affecting the AMI users’ ability to detect modulation (e.g., a neural response that has random temporal variability). However, the TMTF results for AMI users are consistent with a significantly longer TI time window than for CI users based on a higher weighting for the longer time constant and/or an increase in the actual time constants. When using the values of *S* that were fit in experiment 1 at comfortable level, the data for AMI-3 and AMI-5 were broadly consistent with TI window parameters of *w* = 1 and *T*_b2_ of 30 and 100 ms, respectively. These approximate values will be used to represent the possible TI window shape for these subjects in experiments 3 and 4.

## Experiment 3: effect of stimulus duration on detection threshold

### Rationale

Previous published data from AMI users show that, unlike CI users, increasing the rate of stimulation above approximately 250 Hz does not result in a reduction in threshold or level for comfortable loudness (Lim et al. 2008b). This lack of an effect of pulse rate, suggests that the response to AMI stimulation may be subject to greater refractory or adaptation effects than the response to CI stimulation. In this paper, we distinguish between refractory effects (leading to a relatively constant but lower neural response to each pulse after the first pulse in a pulse train, as further explained in the “TI model” section) and adaptation (leading to an overall decreasing neural response during the duration of the pulse train). The possibilities of greater refractory and adaptation effects are consistent with the results of experiment 1, since, if midbrain neurons are being activated at or near their saturation level, there will be little opportunity to evoke additional activity when additional stimulus pulses are included for a higher rate stimulus. In particular, the results of experiment 1 predict that a stimulus with rate above 500 Hz (corresponding to the refractory recovery period of 2 ms in experiment 1) could not evoke additional overall activity over a stimulus of rate 500 Hz. In this experiment, refractory and adaptation effects were investigated by measuring the effect of pulse train duration on thresholds for different pulse rates. Significant adaptation for high rates would be evident as a shorter duration at which thresholds reach a plateau as duration increased compared to that for low rates (where adaptation is not expected to be significant). Refractory effects will influence the slope of the threshold versus rate function, regardless of whether adaptation is also present or not.

### Stimuli and procedures

Stimuli in this experiment were unmodulated pulse trains of differing rates (40, 50, 100, 200, 600, 1,200 Hz) and durations (from a single pulse [AMI-3] or three pulses [AMI-5] to at least 400 ms). Stimulus mode, pulse timing parameters, and electrode position were identical to those used in experiments 1 and 2.

For AMI-5, some additional stimuli with sinusoidal current modulation were used to test the hypothesis that modulation would prevent or limit any neural adaptation during the pulse train with a carrier rate of 1,200 Hz. The modulation frequencies used were 20, 40, and 100 Hz, and a large modulation depth (peak to valley) of 80 CL was used. The peak current was the same as in the unmodulated stimuli.

For each stimulus condition (pulse rate and duration), a 3IFC adaptive task (as in experiment 1) was used to determine the detection threshold. For each stimulus rate, the order of testing of the different durations was pseudo-randomized. Each threshold was measured twice and averaged.

## Results and discussion

**FIG. 7:**
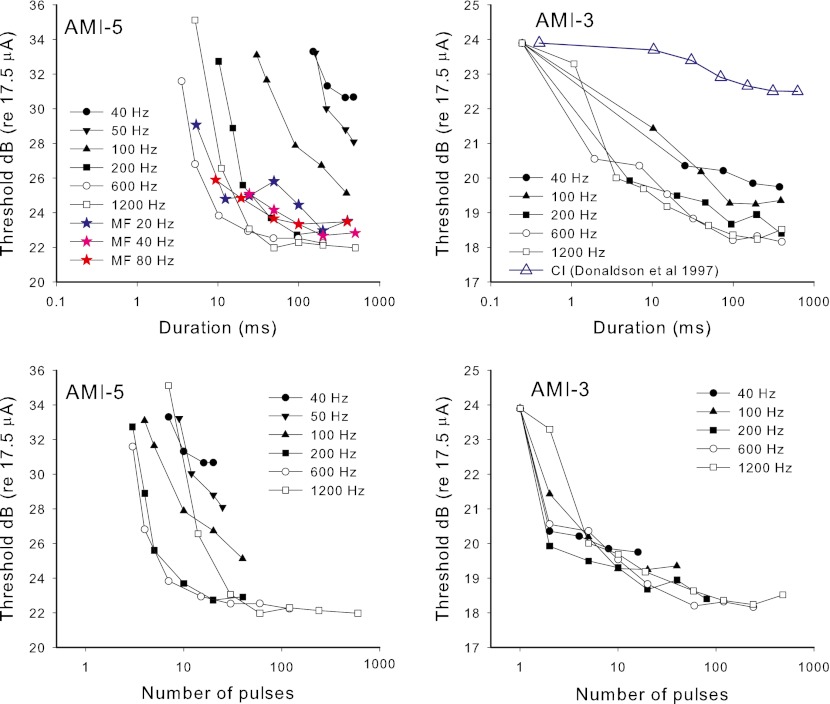
Results of experiment 3. In the *top panels*, detection thresholds are depicted versus the stimulus duration. In the *bottom panels*, the same data are shown as a function of number of pulses in the stimulus. In the *top left panel* (AMI-5), the additional thresholds for modulated pulse trains are also shown (*colored stars*). The *blue crosses* in the *top right panel* show the average CI data for 100-Hz pulse trains from Donaldson et al. (1997).

The thresholds versus stimulus duration for different rates of stimulation are shown in the top panels of Figure 7, and the same data are plotted as a function of number of pulses in the bottom panels. The average CI data from Donaldson et al. (1997) for pulse trains at 100 Hz are shown for comparison in the top right panel. To aid the comparison of CI with AMI data, the absolute levels of the CI data have been adjusted so that the threshold for a single pulse is the same as for AMI-3. The variability of the CI data was small with the standard deviation at the greatest duration being less than one dB (range −1 to 3 dB lower than the single pulse threshold, with the exception of one electrode from one subject). There are no published human CI data for other rates of stimulation. It can be seen that both AMI users had threshold versus duration functions that were much steeper for small durations than the average function of CI subjects. AMI-5 showed very steep functions for rates of 100 Hz or less, with thresholds significantly lowering with increased duration even out to the maximum duration tested. In contrast, for rates of 200 Hz and above, the effect of duration was steep for small durations but plateaued at around 50-ms duration. The difference between high and low rates of the duration at which thresholds plateaued is an indication that adaptation was occurring during the high-rate (≥200 Hz) pulse trains for this subject. The use of modulation with the 1,200-Hz pulse train with AMI-5 did not appreciably change the duration at which the threshold plateaued compared with the unmodulated stimulus, and thus did not affect the apparent adaptation. The thresholds of AMI-3 plateaued for rates of 100 Hz and above at a duration of around 100 ms, which was quite similar to that of the average CI data. Although the 40-Hz thresholds continued to drop slightly for durations greater than 100 ms, the effect was not large enough to say confidently that it differed from the higher rates or the CI data. Thus, there was no clear evidence of adaptation at high rates for AMI-3, at least at threshold.

**FIG. 8:**
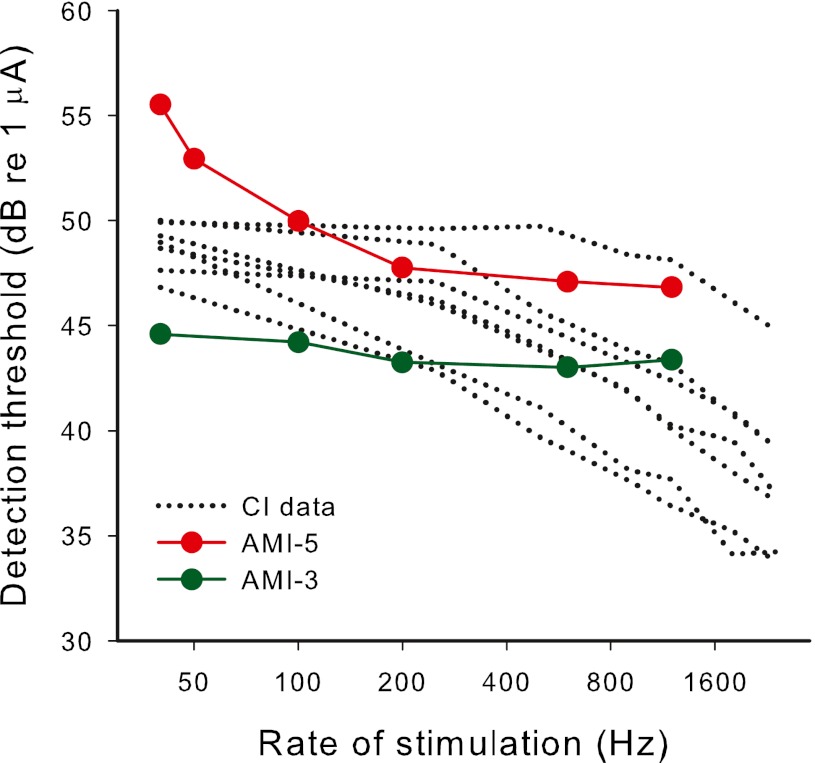
The effect of pulse rate on detection thresholds for pulse trains of 500-ms duration for seven CI subjects (*black dotted lines*) and the two AMI subjects (*filled circles*).

The data for both AMI users exhibit the previously noted limited effect of rate on detection threshold for rates above a certain value. Looking at the thresholds for long durations (i.e., 500 ms) in Figure 7, it is clear that thresholds for low rates differ more than thresholds for high rates. In both cases, the 600- and 1,200-Hz thresholds are essentially equal and the 200 Hz threshold is closer to that of the higher rates than that of 100 Hz. Figure 8 shows threshold versus rate data for both AMI subjects for the longest duration stimuli together with example data from our laboratory for seven CI subjects using pulse trains of 500 ms duration, showing that, in contrast to the AMI users, thresholds for CI users drop more steeply for increases of rate above 250 Hz than for increases of lower rates. Therefore, if experiment 3 were carried out with CI subjects, it would be expected that the functions of threshold versus duration would plateau at long durations with threshold values more similar for low rates than for high rates.

Note that for both AMI users, the threshold versus duration function for the 1,200-Hz stimulus did not generally follow the pattern set by the lower-rate stimuli. This is more evident in the lower panels in Figure 7. For AMI-5, the 1,200-Hz stimulus had a higher threshold than most of the lower rates given a constant small number of pulses and even a higher threshold than the 600 Hz rate for durations less than 20 ms, in which the 1,200-Hz stimulus contained double the number of pulses than the 600-Hz stimulus. For AMI-3, a similar trend was observed at a short duration around 1 ms. This pattern indicates that the effect of rate on the neural response at 1,200 Hz had greater influence than the counteracting effect of TI. For AMI-3, it can be seen that the functions expressed relative to number of pulses, except for the 1,200-Hz rate, collapse approximately onto a single function. Thus, for small durations, the threshold was determined more by the number of pulses in a pulse train than its duration. These data, along with the smaller effect of higher rates on longer-duration thresholds than that observed in CI users, suggest that a larger effect of neural refractory behavior on thresholds is occurring in AMI users compared to CI users. This greater neural refractory effect is consistent with the results of experiment 1 and with the midbrain neurons being activated at or close to their saturation response.

In summary, the results for AMI users indicate that, compared to CI users, there is a greater effect of duration on threshold for short durations. The changes of threshold with rate for both AMI users suggest a greater influence of neural refractory behavior on thresholds than in CI users. For AMI-5, the results were consistent with an additional influence of adaptation occurring at rates of 200 Hz and above.

### TI model

For this experiment, it was assumed that the threshold for each rate and duration corresponded to a criterion TI window maximum output, as in experiment 1. The effect of rate on neural excitation for each pulse in the pulse train was first modeled as a constant lower excitation for every pulse except the first pulse (E1), with the reduction increasing as rate increased: that is, a refractory effect that increased with rate was included, but initially with no adaption. This simple model follows what is seen in ECAP studies (Hughes et al. 2012), but, as a first step, ignores the effect of adaptation (further reduction in response during the pulse train) and also ignores the alternation of ECAP response amplitude for successive stimulus pulses seen at some rates. First, the 500-ms-duration data were used to derive the reduction of excitation relative to that for the first pulse (E1) as a function of rate. The threshold versus rate data were fit by adjusting the excitation reduction versus rate function (and hence the excitation values for each pulse at the input to the TI window for different rates) in such a way as to correctly predict the stimulus level change needed to maintain the same criterion TI window maximum output for higher rates as for the 40 Hz stimulus. Thus the fitting parameters were the excitation reduction versus rate function and the relation between current and excitation (parameter *S*).

**FIG. 9:**
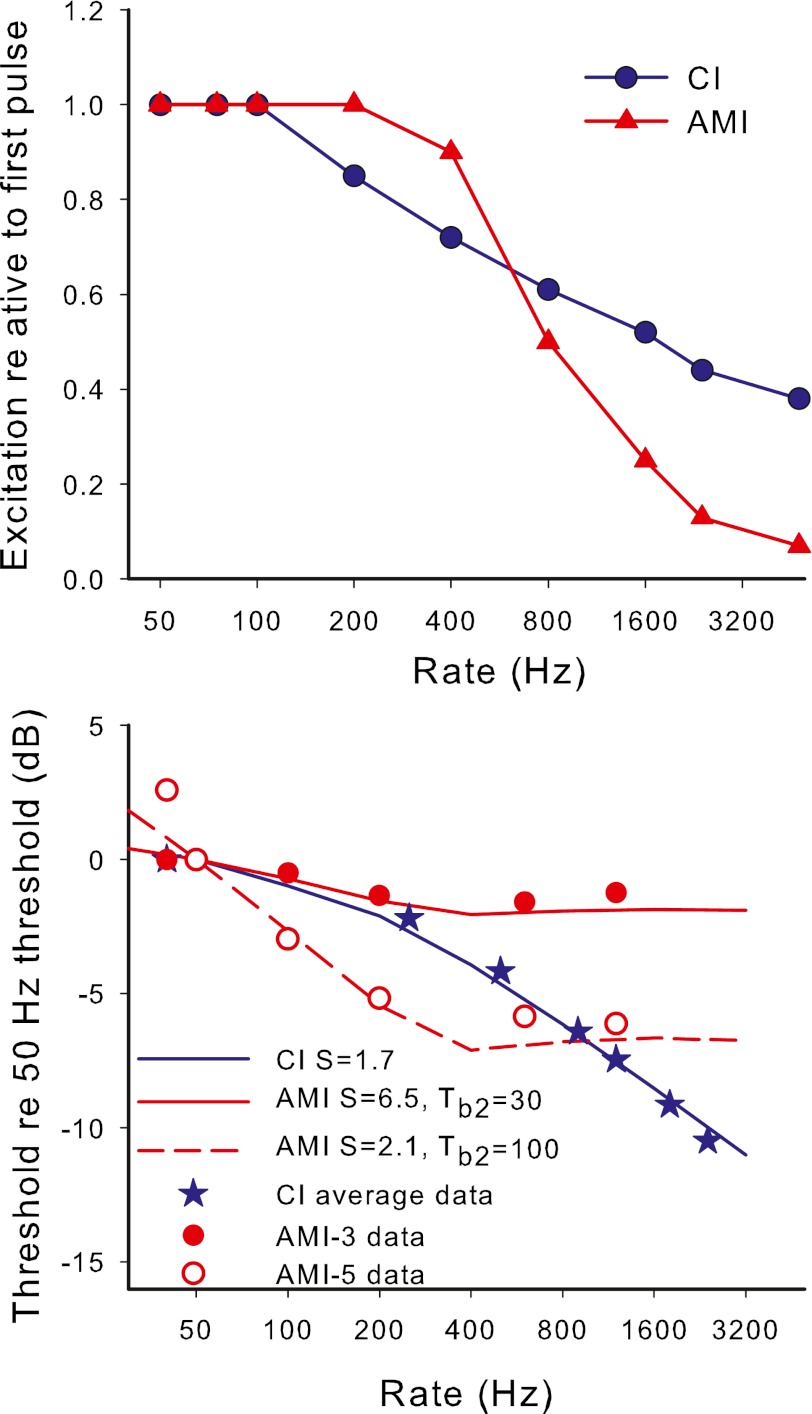
*Top panel*: modeled neural excitation evoked by the second and subsequent pulses in a pulse train relative to the first pulse as a function of pulse rate. *Blue circles* are for CI users and red triangles are for AMI users. Each function was fitted with a sigmoid function for input into the TI model. *Bottom panel*: *lines* show the modeled detection thresholds relative to 50 Hz for long duration (500 ms) pulse trains for different pulse rates, using the functions shown in the *top panel*. Symbols are actual data for CI (averaged from Fig. 8) and for each AMI subject.

For CI users, the average threshold versus pulse rate data calculated from Figure 8 were modeled. The fitted input function is shown in the top panel of Figure 9 (blue filled circles). The average CI data from Figure 8 are shown in the bottom panel of Figure 9 (blue stars) with the fitted model predictions for 500-ms duration thresholds (blue line). The fitted output transform parameter (*S* = 1.7) is reasonably close to the average value at threshold (*S* = 1.45) derived by McKay and McDermott (1998) from a different group of CI users.

For AMI subjects, the function of reduction in neural response relative to the first pulse versus pulse rate was fitted to be consistent with the effect of rate on the 500-ms-duration thresholds of AMI-3 and AMI-5. The TI window parameters used in this fitting procedure were those that were consistent with the data from experiment 2, that is, *w* = 1 and *T*_b2_ equal to 30 and 100 ms for AMI-3 and AMI-5, respectively. The fitted function of reduction of neural response versus rate is shown in the top panel of Figure 9 (red triangles). It can be seen that, in comparison to the CI function, the AMI function needed to be steeper to predict a flat rate versus threshold function for rates above 400 Hz. That is, as rate increased in the higher-rate range, the drop in excitation per pulse was balanced by the increase in number of pulses integrated in the TI window, leading to a constant maximum window output and no required stimulus level adjustment. The wider TI windows for AMI users, particularly for AMI-5, were needed to predict the steeper effect of rate on threshold at low rates compared to CI users. The solid and dashed red lines in the bottom panel of Figure 9 are model predictions that correspond approximately to the data of AMI-3 (closed circles) and AMI-5 (open circles), respectively. The fitted output transform parameter for AMI-3 (*S* = 6.5) was consistent with that derived from the threshold data of experiment 1, when ignoring the transient peak at the short IPI. The fitted value of *S* used for AMI-5 was higher than that fitted for the threshold data in experiment 1 (2.1 compared to approximately 1.3, respectively). This may be because the model omitted the effect of adaptation, which was occurring here but not at the low rate (100 Hz) used in experiment 1. As mentioned in the “Results” section, the fact that the thresholds plateaued at a smaller duration for high rates for AMI-5, suggested that adaptation was occurring for rates of 200 Hz and above, in addition to the refractory effect. Adaptation would make the slope of the threshold versus rate function less steep than it would have been without adaptation (leading to a higher apparent *S* if not taken into account).

**FIG. 10:**
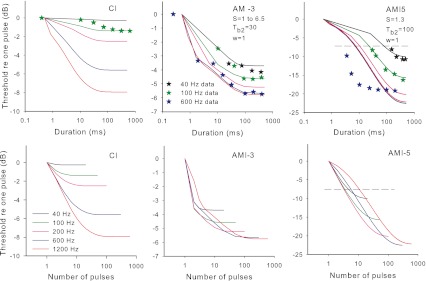
Model predictions for the effect of duration on detection threshold for different pulse rates. The *top panels* show the predictions for typical CI, AMI-3 and AMI-5, and the *bottom panels* show the same data plotted as a function of the number of pulses. The modifications to the TI window for AMI subjects are shown in the *top panels*. Both the AMI models used the excitation versus rate function in Figure 9 (*top panel*). The *dotted lines* in the AMI-5 panels show the approximate limit above which actual data for AMI-5 could not be measured. The CI data for 100 Hz, and the AMI data for 40, 100, and 600 Hz are re-plotted for easier comparison.

Using the same fitted parameters as derived above, the predicted effects of duration for the different rates were calculated by determining the stimulus level change required to produce the same maximum TI window output as the first stimulus pulse on its own. Figure 10 shows the predicted thresholds relative to that for a single pulse, with the top panels showing thresholds as a function of duration and the bottom panels showing thresholds as a function of number of pulses. Since the thresholds for different durations varied in level over a large range for the AMI users, it was necessary to examine whether use of a constant *S* value across the durations (hence levels) was appropriate. If the AMI data and model from experiment 1 are examined (Figs. 2 and 4) it is apparent that the effect of IPI was smaller at threshold than at comfortable level, implying a larger *S* at threshold than at comfortable level. For AMI-3 there was a very large effect of level in experiment 1, implying a large change in *S* (6.5 to 1.45) between the threshold and comfortable level of a 200-Hz pulse train (a dynamic range of 4.7 dB). Since the single-pulse threshold of AMI-3 in experiment 3 was slightly higher than the comfortable level for the 200-Hz pulse train in experiment 1, the *S* values used for the model prediction in Figure 10 started with 1 at the highest level to predict the change in threshold from one to two pulses, then used a higher *S* value to predict the change from two to five pulses, and so on, increasing *S* smoothly to 6.5 to predict changes in threshold for long-duration reference thresholds. For AMI-5, the effect of level in experiment 1 was small, and so, for simplicity, a constant *S* value was used in the predictions in Figure 10. Given the discussion above about the discrepancy in *S* values for this subject, the predictions in Figure 10 used the fitted value from experiment 1 (*S* = 1.3) with the expectation that the predictions would hold only for the rates lower than 200 Hz, for which adaptation was not expected. For CI users, in contrast to the AMI users, *S* tends to increase at high absolute current levels (McKay and McDermott 1998). However, since the range of thresholds for different durations is relatively small, at least for 100-Hz pulse trains, this effect was ignored and a constant *S* value of 1.7 as fitted above was used.

It should be noted that, for simplicity, any effect of multiple looks has been ignored in this model. The TI window output increases with time from stimulus onset for a time that depends on the window time constants, and decreases near the end of the pulse train. Therefore, the maximum window output occurs only for a portion of the stimulus duration (only at the final pulse for AMI-5, because of the long integration window, and for short durations for CI users and AMI-3). The application of multiple looks (the window output at each pulse position being a ‘look’) would require a complex weighting of looks across the stimulus. In contrast, the multiple looks used in experiment 2 represented modulation periods for which the OMD was relatively constant across the stimulus.

The predicted detection thresholds for CI users and AMI-3 generally follow the pattern shown by the data in Figure 7. The predictions for CI data at 100 Hz show a magnitude and pattern consistent with the typical CI data shown by Donaldson et al. (1997). In the top panels of Figure 10, for AMI-3 and AMI-5, the data for 40, 100, and 600 Hz are re-plotted for comparison with the model. It can be seen that the data for AMI-3 and the low-rate data (40 and 100 Hz) for AMI-5 fit the model reasonably well. As expected the model does not predict the higher-rate data of AMI-5 well. The thresholds drop more steeply and plateau at a much earlier duration than predicted by the model without adaptation. The difference between model predictions and data suggests that the excitation per pulse evoked by the higher-rate pulse train for AMI-5, in contrast to our simple refractory model, continues to decrease over the whole duration of the pulse train, starting at higher values than the average fitted values in the top panel Figure 9. By 50 ms after the start of the pulse train, the excitation per pulse is sufficiently low so that further increases in duration do not increase the TI window output and thus do not change the threshold.

## Experiment 4: forward masking

### Rationale

This experiment investigated recovery from forward masking for AMI compared to CI users. Oxenham and Moore (1994) and Plack et al. (2002) have shown that the probe threshold changes with masker-probe offset in forward masking can be modeled by the effects of a TI window. In this model, the decay of masking is assumed to be due to a persistence of activity due to the masker after its offset rather than a recovery of neural activity due to the probe (Oxenham 2001). For a fixed masker and probe duration, the probe threshold shifts are most sensitive to the long-duration time constant in the TI window (*T*_b2_). Experiments with CI listeners have shown temporal forward masking functions that have a similar shape to normally hearing listeners (Shannon 1990; Chatterjee 1999; Nelson and Donaldson 2002).

### Stimuli and procedures

**FIG. 11:**
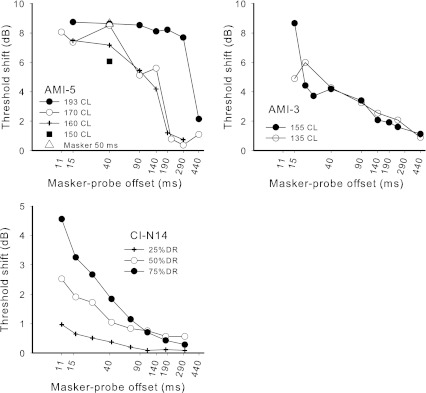
Results of experiment 4, showing probe threshold shift in dB as a function of masker-probe offset (offset-to-offset). Masker duration was 75 ms, except for one data point for AMI-5 (*open triangle*) where it was 50 ms. Probe duration was 10 ms. Masker and probe were pulse trains of 1,200-Hz rate. The *bottom panel* shows representative CI data re-plotted from Nelson and Donaldson (2002) in the same units as the AMI data. Masker and probe were 500-Hz pulse trains with durations of 320 and 10 ms, respectively.

The probe and masker stimuli were both pulse trains of 1,200 Hz with durations of 10 and 75 ms, respectively. The silent gap between masker offset and probe onset varied between 1 and 430 ms. Nelson and Donaldson (2002) proposed that the appropriate measure of masker-probe offset is the offset-to-offset time, since the final pulses of the probe determine whether the probe is heard or not, and we follow this offset notation. For small offsets, subjects may have difficulty distinguishing the masker and probe, leading to overestimation of the decay of masking (McKay 2012). To limit this possibility, different low rates of stimulation could be used to perceptually differentiate the masker and probe, or the minimum offset (including probe duration) could be greater than 10 % of the masker duration (Lapid et al. 2008), to ensure that a duration cue is likely for all offsets. Since we were interested in the forward masking effect at high rates, we chose the second option. However, it should be noted that duration discrimination was not tested in the AMI subjects, so it is unclear whether this maneuver would have been useful in practice. For both AMI users, two or more masker current levels (see Fig. 11) were used to evaluate growth of masking with masker level.

Masked and unmasked probe thresholds were determined using an adaptive 3IFC procedure. For unmasked thresholds, the procedure was identical to that in experiment 3. For masked thresholds, two reference intervals contained the masker only, and the subject was asked to identify which interval contained the masker + probe stimulus (or the ‘different’ stimulus).

For AMI-5, an additional data point was collected at the higher masker level using a masker of 50 ms duration, gap of 30 ms, and probe duration of 10 ms (40-ms masker-probe offset). Comparison of this threshold with that following the 75-ms masker with 40 ms masker-probe offset determined the effect of masker duration while keeping the offset the same. Comparison of the additional threshold with the 75-ms masker and 15-ms offset investigated whether the final 25 ms of the 75-ms masker contributed to the masking of the probe (i.e., the 50-ms masker condition replaced the final 25 ms of the 75-ms probe with silence). This second comparison thus investigated whether the response to the 75-ms masker had significantly adapted by 50 ms after its onset.

## Results and discussion

Figure 11 shows the forward masking functions for AMI-3 and AMI-5. For comparison, the lower panel shows typical forward masking functions for a CI subject, re-plotted from Nelson and Donaldson (2002) using the same measurement units. It can be seen that the shape of the functions for AMI-3 were relatively consistent with those of the CI subject, unlike those for AMI-5, which showed the largest decreases in threshold as the offsets increased above about 140 ms. One major difference between the functions for AMI and CI subjects is the effect of masker level. For both AMI subjects, most of the data for different masker levels, except for the highest level for AMI-5, overlaid each other, resulting in no growth of masking with masker level. At first sight, the lack of masking growth is difficult to understand, given that the different masker levels certainly evoked different loudness percepts. However, it could be consistent with a degree of adaptation throughout the masker pulse train. In forward masking, the amount of masking following masker offset is dependent on the output of the integration window when centered on the *offset* of the masker (as well as the TI window shape, which determines the rate of masking decay from that point). However, the loudness of the masker is related to the *maximum* TI window output, which may occur when the center of the window is closer to the *start* of the pulse train. If there is neural adaptation occurring at times following the time of maximum output, then the window output at the end of the pulse train could be significantly lower than the maximum output. If the degree of adaptation increased with masker level, the *maximum* window output (and therefore loudness) may increase with level while the output at the *end* of the pulse train remained relatively constant, resulting in no growth of masking. Although AMI-3’s data did not show adaptation in experiment 3 at threshold level for the 1,200-Hz pulse train, it is possible that adaptation occurred at the higher levels used for the masker in this experiment.

The masked probe threshold after the 50-ms masker followed by a gap of 30 ms (215.3 CL: open triangle in Fig. 11) for AMI-5, was essentially equal to that after the 75-ms masker followed by a 5-ms gap (216 CL), demonstrating that the final 25 ms of the 75-ms masker had little influence on the probe threshold, and supporting the existence of significant adaptation during the masker. The 50-ms masker with 30-ms gap also produced the same masking as the 75-ms masker with 30-ms gap (215.3 CL). This lack of masker duration effect may be a reflection of the lack of masking decay seen at this current level for a lengthy period after masker offset (up to 300 ms), together with the adaptation.

### TI model

In the TI model of forward masking, probe thresholds corresponded to a criterion maximum difference (in dB) between the outputs of the TI window for the maker-alone and masker + probe conditions (which occurred near the offset of the probe stimulus). In the model the criterion value was set at 3 dB (a typical value obtained from studies with normal hearing). The probe stimulus level was adjusted for different probe offsets to maintain a constant 3 dB ratio at time of maximum window output difference. To model the unmasked probe threshold (and hence threshold shift) a level of internal noise was introduced so that the criterion window output when the probe was present was 3 dB higher than that for either the masker alone or the internal noise. The same TI window parameters and *S* values that were used for experiment 3 were used in this experiment. That is, predictions were made based solely on the parameters found in the previous experiment, initially disregarding adaptation. For AMI-5, a simple adaptation model was then included, whereby the neural responses to the second and subsequent pulses of the masker were arbitrarily linearly reduced from the level predicted in Figure 9 (top panel) to zero for the last pulse.

**FIG. 12:**
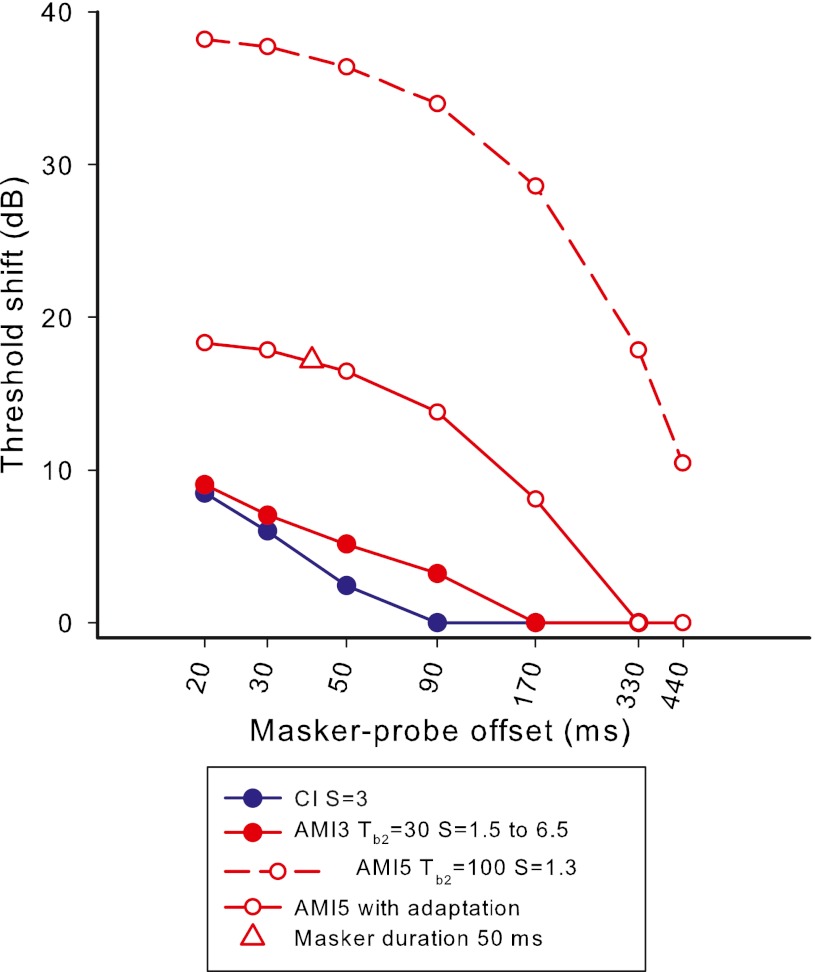
Predicted forward-masked thresholds for CI users (*blue*) and AMI users (*red*). The *closed red symbols* are the predictions that use the previous model parameters for AMI-3 and the *open symbols* are for AMI-5. The *dashed and solid lines* for AMI-5 show the effect of not including or including some adaptation to the model of excitation from the masker, respectively.

Figure 12 shows the predicted forward-masked threshold shifts for CI and AMI users. It can be seen that the CI and AMI-3 predictions show a roughly linear decay of masking (on log–log scales) with the AMI-3 prediction showing a slower decay of masking due to the lengthened TI window. The model predictions for AMI-5 without adaptation show a very slow decay of masking that is nonlinear, similar to the measured data. However, the predicted amount of threshold shift is very large compared to the actual values in Figure 11. The predicted masker TI window output for AMI-5 without adaptation (red dashed line) is much larger than for AMI-3 because the TI window is integrating over a longer time, resulting in greater masking. When adaptation is included in the model (red solid line) the TI window output at the offset of the masker is significantly reduced. Furthermore, with adaptation, the window output does not increase so steeply as the center of the window moves along the masker stimulus, so that the output at the masker offset is much less sensitive to masker duration. It can be seen in Figure 12 that the inclusion of adaptation for AMI-5 significantly improved the model predictions, but even more adaptation than the simple linear model used here would be necessary to reduce the masking to the values of the measured data.

## General discussion and conclusions

### Model considerations

For typical CI users, the effects of temporal parameters on loudness and detection thresholds across the four experiments (interpulse interval, modulation, duration, and masker-probe offset) could successfully be modeled by fitting appropriate functions that define neural excitation evoked by each stimulus pulse together with the slope of the excitation versus current function. Furthermore, the same TI window parameters as derived from normally hearing listeners were successfully used in the model fitting for CI users. This is a phenomenological model that ignores some of the details of auditory temporal processing and psychophysical perception, and there are many aspects of hearing, such as perception of fine temporal structure, that cannot be modeled in such a simple way. However, the fact that CI and normal hearing perception can be modeled with the same central processing parameters indicates that AN activity is likely to be an appropriate input into the TI window in each case, as proposed by Plack et al. (2002), and that central temporal processing of AN activity is largely unaffected in CI users by peripheral hearing loss or electrical stimulation of the AN, at least for the perceptual tasks investigated here (see also “Neural mechanisms of temporal integration” section).

To adequately describe the AMI users’ data, both the input function (describing neural activity input to the TI window) and the TI window parameters themselves differed from those used for CI users. There were many potential parameters in each experiment, which made it difficult to independently fit each accurately. However, the data from each experiment were sensitive to the effects of different parameters, allowing an overall picture to be built of how the parameters must be changed from those applied to CI data to describe all the AMI experiments when viewed together.

Experiment 1 showed that, unlike in CI users, the second of two pulses did not contribute to loudness for very short interpulse intervals, suggesting that the midbrain neurons were activated more deterministically than AN neurons (i.e., all the available midbrain neurons were activated by the first pulse and were not available for the second pulse until the end of their refractory period). The modeled response recovery time was very short compared to the modeled recovery for CI users, consistent with neurons deterministically activated well above their individual thresholds. These neural properties were consistent with the neural response versus rate function in experiment 3 that was steeper than that for CI users (Fig. 9, top panel), and was needed to explain the lack of effect of rate on loudness for higher rates. The neural adaptation that was evident for AMI-5 for high rates in experiment 3 was consistent with adaptation needed for the same subject to limit the predicted amount of forward masking in experiment 4.

Experiments 2, 3, and 4, were all consistent with a greatly lengthened TI window in AMI users compared to CI users, which explained the poor temporal resolution (experiment 2), the large effects of low pulse rates and short durations on detection thresholds (experiment 3), and the slower recovery from forward masking (experiment 4). Although the TI window parameters could not be accurately fitted in each experiment, the approximate time constants of 30 and 100 ms for AMI-3 and AMI-5 (compared to approximately 7 ms for the dominant part of the CI and normal hearing window) consistently described the data in all three experiments as well as the differences between the two AMI users.

The slopes of the current-to-excitation functions (*S*) fitted for the data in experiment 1 differed across the three AMI subjects, but the range of values was consistent with that across a group of CI users (McKay and McDermott 1998). One difference seen compared to CI users was that the slopes decreased rather than increased with increasing level. The increase of *S* with increasing level in CI users is probably related to the higher density of neural material as distance is increased from the intracochlear electrodes. For low currents, sparsely distributed cell bodies in the spiral ganglion (and any surviving peripheral dendrites) are activated, whereas for higher currents, more densely packed axons in the central auditory meatus may be activated (van den Honert and Stypulkowski 1984). For the shank electrodes used in the AMI device, in contrast, the stimulated neural material is very close to the electrode site and may not vary in density as much with distance. The fact that values of *S* consistent with the ones fitted in experiment 1 could be used to fit the data in the other three experiments supports the consistency of the model in describing the results of the various experiments.

Although the AMI users exhibited the same general pattern of results across the experiments, there were some important differences that may be related to the site of the electrodes. AMI-3 was implanted in the desired target (ICC), whereas the others were implanted somewhat outside this area. AMI-3 showed temporal resolution ability that was less impaired compared to CI users than was the poor resolution ability of AMI-5, with a fitted TI window that was closer in width to that of the CI users. Although both AMI users’ data were consistent with a steeper rate versus neural response function (Fig. 9, top panel), and the presence of adaptation for above-threshold 1,200-Hz stimuli (experiment 4), AMI-5’s data implied significantly more adaptation and at lower rates in experiments 3 and 4. The slope of the current-to-excitation function (*S*) for AMI-3 was very steep near threshold and decreased with level (experiment 1, and needed for experiments 3 and 4), whereas the slopes for AMI-5 and AMI-2 decreased with level by a much smaller amount. This latter difference could potentially be due to the closer proximity of the desired neural targets in the ICC in AMI-3.

### Neural mechanisms of temporal integration

Although the model successfully predicted the measured AMI data, the results do not necessarily relate to the neural mechanisms associated with midbrain activity or central neural processing of this activity. It is more appropriate to state that the AMI model represents what would occur in CI users if they had experienced those changes to the inputted neural activity and TI window shape. Thus, AMI users *behave as if* their TI window is wider than that of CI users in addition to having stronger refractory effects on the inputted activity. These modeling results help to conceptualize the types of perceptual difficulties encountered by AMI users, especially since they currently use the same stimulation strategies as CI users.

There are several potential physiological explanations for the altered temporal window seen in the AMI data: the integration mechanism may partly lie at centers below the midbrain; the integration mechanism may be dysfunctional because of plastic or other effects of peripheral pathology; or the mechanism may not be accessed by the particular stimulus pattern we applied to the midbrain neurons. Gerken et al. (1991) have suggested that the TI mechanism is at or higher than the IC, and also that peripheral hearing loss causes changes to central function. They measured threshold versus duration functions for trains of cathodic monophasic pulses in five cats implanted in the ICC or CN. The cats were tested both before and after a partial cochlear hearing loss was induced by noise exposure. They found that the electrical thresholds decreased and the slopes of the threshold versus duration functions also significantly decreased after noise exposure. Since similar decreases in slope were also seen using acoustic stimulation after induced cochlear hearing loss, it was deduced that the integration mechanism resides at or above the ICC. The mechanism driving the influence of the peripheral hearing loss on the hypersensitive ICC or CN response (lower thresholds) remained unclear. However the smaller slope of threshold versus duration, together with the accompanying reduced variance of the thresholds across repeated measures, is consistent with a steeper current-to-neural excitation function at the central site of stimulation following acute cochlear damage. Similar homeostatic regulation of neural activity in the CN and IC in the presence of cochlear hearing loss has been modeled as a cause of tinnitus (Schaette and Kempter 2006). The results do not necessarily imply that the central integration mechanism itself is affected by the peripheral loss, as the results can be explained by a hypersensitive response to the CN or IC neurons (i.e., change to the input of the integrator). We do not know how the thresholds of our AMI subjects would differ if they had normal cochlear function and an intact AN, but the slopes of the thresholds versus duration for the lowest pulse rates (the closest to the stimuli used by Gerken et al.) had much greater slopes and different shapes than those from the cats with impaired-cochlear function in the study of Gerken et al. There are many differences between this study and the current one, including a species difference, use of monophasic pulses, and partially hearing acutely deafened animals.

The effect of cochlear health on central processing has also been studied in guinea pigs with CIs (Middlebrooks 2004; Kang et al. 2010; Pfingst et al. 2011). In general the findings showed that animals with deafened cochleae (absence of hair cells and dendrites and reduced SGC density) had higher thresholds to pulse trains and the slopes of the threshold versus rate functions below 1,000 Hz were flatter compared with animals that had remaining cochlear function. Although the differences in the threshold versus rate slope can be attributed to the effect of remaining cochlear structures and/or better spiral ganglion cell survival, the data, again, do not necessarily indicate that the central integration mechanism itself is affected by the state of cochlear health. The effect of cochlear health can be modeled as influencing the current-to-neural response function at the level of the cochlea. The flatter threshold versus rate slope for rates below 1,000 Hz is consistent with the slope of current to neural excitation function becoming steeper at higher current levels, due to activation of more central and tightly packed neural material in the internal auditory meatus in animals with poor SGN survival. This observation is consistent with the behavior of loudness growth functions in humans, which have a steeper slope above a certain kneepoint current (McKay et al. 2003). Both the animal and human CI studies show an imperfect relationship, however, between absolute low-rate threshold and slope of the threshold versus rate function (see Fig. 8) and this can be attributed (particularly in humans) to the additional influence of the distance of the electrode from the spiral ganglion or internal auditory meatus.

The animal studies discussed above suggest that the TI mechanism exists at or above the ICC and should still be relatively functional in patients with hearing loss. However, our human AMI data seem to suggest that the integration mechanism, particularly for the short integration window, is not functional for these patients, and that, at least for AMI-5, the long integration window is significantly longer than in the normal case. Given the fact that the CI data were consistent with a normal integration mechanism, the explanation is unlikely to lie in the effects of cochlear health on central mechanisms. An alternative explanation has been provided by recent neurophysiological results in guinea pigs that were stimulated in the ICC with the AMI (Calixto et al. 2012). In that study, a single site within a given frequency lamina of the ICC was stimulated with a pair of electrical pulses with varying interpulse intervals and the corresponding neural activity was recorded in the primary auditory cortex of a similar frequency region. It was shown that, at very short intervals (<2 ms), significant or complete refraction occurred for activity to the second pulse, consistent with the data in Figure 2. Furthermore, for longer intervals (up to 100 ms), the activity to the second pulse did not fully recover to the level of the first pulse and was significantly reduced compared to what has been observed for paired acoustic click stimuli (Eggermont and Smith 1995; Wehr and Zador 2005). However, by stimulating two different sites in the same isofrequency lamina, each with a single pulse, this strong refractory and suppressive effect was avoided. These findings support the strong refractory and adaptation effects observed in the AMI users in the current paper in response to repeated stimulation of the same electrode site. Additionally, Calixto et al. showed that it was possible to achieve enhanced cortical activity to short (<2 ms) interpulse intervals, consistent with the effect of a narrow TI window of the shape and duration used to model CI data here, by stimulating two different sites along an isofrequency lamina. This suggests the possibility that the narrow TI integration window may be coded within centers higher than the IC, but current AMI users are unable to access it due to strong refractory effects caused by artificial activation of a single IC site within each isofrequency lamina. This is further supported by numerous animal studies demonstrating that faster temporal features become coded more as a rate and/or population code across central neurons in higher auditory centers (Frisina 2001; Joris et al. 2004; Wang et al. 2008), and thus stimulation of more than one site may be required to achieve sufficient transmission of temporal cues.

### Clinical considerations

Poor temporal resolution and increased forward masking are both likely to impair speech perception. Envelope modulation in speech signals conveys information about the manner of articulation and voicing in consonants, vowel duration information and voice pitch information. Slow decay of forward masking is likely to produce masking of one phoneme that follows another in the speech stream, and interfere with the segmentation of words and syllables. The poor temporal resolution shown by both AMI-3 and AMI-5 is likely to underlie much of their difficulty with speech understanding.

Following the results of Calixto et al. (2012), a new design of AMI electrode arrays in which activation can occur across different places in each isofrequency lamina may provide a way to improve the temporal resolution of AMI users as well as to minimize refractory and adaptive effects. Additionally, optimized outcomes might be obtained by careful adjustment of electrical stimulus parameters (such as rate and modulation depth) to avoid deleterious effects of adaptation, while maintaining temporal information transmission ability. Given the possible relationship between the extremely poor temporal resolution of AMI-5 and the poor electrode placement in that subject, improved surgical techniques that allow accurate placement of electrodes in the ICC would appear to be an important goal of further clinical research.

## Conclusions

The TI model was successful in describing the temporal processing characteristics of CI users, using the same central processing of AN activity as normally hearing listeners. In contrast, the AMI users showed very poor temporal resolution ability, consistent with a greatly widened TI window, together with a presumed midbrain neural response to electrical stimulation that has increased refractory and adaptation characteristics compared to the AN neural response for CI users.
